# Distinct surfaces on Cdc5/PLK Polo-box domain orchestrate combinatorial substrate recognition during cell division

**DOI:** 10.1038/s41598-020-60344-4

**Published:** 2020-02-25

**Authors:** Ahmad W. Almawi, Laurence Langlois-Lemay, Stephen Boulton, Javier Rodríguez González, Giuseppe Melacini, Damien D’Amours, Alba Guarné

**Affiliations:** 1 Department of Biochemistry and Biomedical Sciences, McMaster University, Hamilton, ON Canada; 2 Ottawa Institute of Systems Biology, Department of Cellular and Molecular Medicine, University of Ottawa, Ottawa, ON Canada; 3 Department of Chemistry and Chemical Biology, McMaster University, Hamilton, ON Canada; 4 Department of Biochemistry, McGill University, Montreal, QC Canada; 5 Present Address: IniXium, 500 Boulevard Cartier Ouest, Laval, QC Canada

**Keywords:** Mitosis, Solution-state NMR, X-ray crystallography

## Abstract

Polo-like kinases (Plks) are key cell cycle regulators. They contain a kinase domain followed by a polo-box domain that recognizes phosphorylated substrates and enhances their phosphorylation. The regulatory subunit of the Dbf4-dependent kinase complex interacts with the polo-box domain of Cdc5 (the sole Plk in *Saccharomyces cerevisiae*) in a phosphorylation-independent manner. We have solved the crystal structures of the polo-box domain of Cdc5 on its own and in the presence of peptides derived from Dbf4 and a canonical phosphorylated substrate. The structure bound to the Dbf4-peptide reveals an additional density on the surface opposite to the phospho-peptide binding site that allowed us to propose a model for the interaction. We found that the two peptides can bind simultaneously and non-competitively to the polo-box domain in solution. Furthermore, point mutations on the surface opposite to the phosphopeptide binding site of the polo-box domain disrupt the interaction with the Dbf4 peptide in solution and cause an early anaphase arrest phenotype distinct from the mitotic exit defect typically observed in *cdc5* mutants. Collectively, our data illustrates the importance of non-canonical interactions mediated by the polo-box domain and provide key mechanistic insights into the combinatorial recognition of substrates by Polo-like kinases.

## Introduction

Processes that drive mitotic progression are under strict cellular regulation to ensure the faithful propagation of newly replicated genetic material. Cellular defects that arise during mitosis – such as sister chromatid mis-alignment or spindle pole mis-positioning – can lead to chromosome segregation defects and give rise to polyploid and aneuploid daughter cells^1–3^. It is during these events that the cell activates signaling cascades known as checkpoints to inhibit mitotic processes^4^. Misregulation of mitotic checkpoints gives cells a proliferative advantage – one of the hallmarks of carcinogenesis^3,5^.

A conserved family of kinases known as Polo-like kinases (Plks) control mitotic events^6^. Plks function in mitotic entry, spindle pole dynamics, chromosome condensation^7^, sister-chromatid segregation^8^, and cytokinesis^2,6,9^. Plk1 is upregulated in multiple human tumors and, therefore, has become an attractive anti-cancer target^10,11^. The structural similarity with other cellular kinases, however, has limited the potential of inhibiting Plk1 due to off-target effects. Some of the most selective drugs against Plk1 also inhibit Plk2 and Plk3 with similar potency^12,13^. Therefore, targeting interactions that regulate kinase activity provide a promising approach to specifically target Plk1.

Much of our understanding of Plk1 comes from Cdc5, the sole Plk in budding yeast^4,14,15^. Cdc5 promotes the release of Cdc14 from the nucleolus and regulates the mitotic exit network (MEN)^9,16–18^ as well as cytokinesis^19–21^. Full release of Cdc14 leads to the downstream activation of the anaphase promoting complex, cyclin destruction, mitotic spindle disassembly, and displacement of the septin ring during cytokinesis^16,18,22^. Cdc5 is inhibited during the spindle position checkpoint (SPOC), which is activated in response to mis-aligned and/or damaged spindle poles^23^. One of the components of SPOC is the Dbf4-dependent kinase (DDK) complex^23^. DDK is a heterodimer formed by the association of the Cdc7 kinase and its regulatory subunit, Dbf4, necessary at multiple steps of the cell cycle^23–26^. During SPOC, Dbf4 binds to Cdc5 and promotes Cdc7-mediated phosphorylation of Cdc5, which then presumably prevents Cdc5 from recognizing its substrates in the MEN pathway^23,27^. However, DDK can also activate Cdc5^28,29^. Cdc5, along with DDK and CDK1, hyper-phosphorylate the structure-specific nuclease Mus81-Mms4 and this, in turn, activates Mus81-Mms4 to resolve joint DNA molecules at the onset of mitosis^29^. Full activation of Mus81-Mms4 depends on the Cdc5-Dbf4 interaction, indicating that DDK and Cdc5 work together during this process^29^.

Like Plks, Cdc5 contains a Ser/Thr kinase domain followed by a polo-box domain that mediates substrate recognition and sub-cellular localization^10,30^. The polo-box domain is made up of two polo boxes that together define the p(S/T)-binding pocket. Polo-box domains recognize substrates harboring a X-S-p(S/T)-(P/X) consensus phosphorylated site^30,31^. However, polo-box domains can also mediate phosphorylation-independent interactions^27,32,33^. For instance, the drosophila microtubule-associated protein Map205 interacts with the polo-box domain of Polo (the drosophila homolog of Plk1) at the pS/T-binding pocket^33^, despite lacking the consensus phosphorylated motif. Dbf4 also lacks the phosphorylated consensus sequence. Instead, it uses a unique polo-interacting motif (^83^RSIEGA^88^) to interact with the polo-box domain of Cdc5^27^. Mutation of key residues at the pS/T-binding pocket does not affect Dbf4 binding, indicating that Dbf4 binds a different surface of the polo-box domain^23,30,31^. However, the surface of the polo-box domain where Dbf4 binds is not known. To understand how Dbf4 binds and modulates the function of Cdc5, we solved the crystal structures of the polo-box domain of Cdc5 on its own and in complex with peptides derived from the Cdc5-binding motifs of Dbf4 and the spindle pole-body protein Spc72 – a bona fide Cdc5 phosphorylated substrate. As expected, the Spc72 peptide bound to the groove at the interface of the two polo boxes. The structure in the presence of the Dbf4 peptide was obtained at lower resolution, but it clearly showed additional electron density on a surface of the polo-box domain opposite to the phosphopeptide binding site. Point mutations on this surface of Cdc5 abrogated binding to the Dbf4 peptide, confirming that the extra density did indeed correspond to the Dbf4 peptide. Mutations on this surface of the polo box domain also result in early anaphase arrest phenotypes distinct from the mitotic exit defect typically observed in *cdc5* mutants. Based on these results, we propose a model for how binding to Dbf4 may help release the auto-inhibition of the Cdc5 kinase.

## Results

### Yeast Cdc5 recognizes phosphorylated substrates like human Plk1

**Figure 1:**
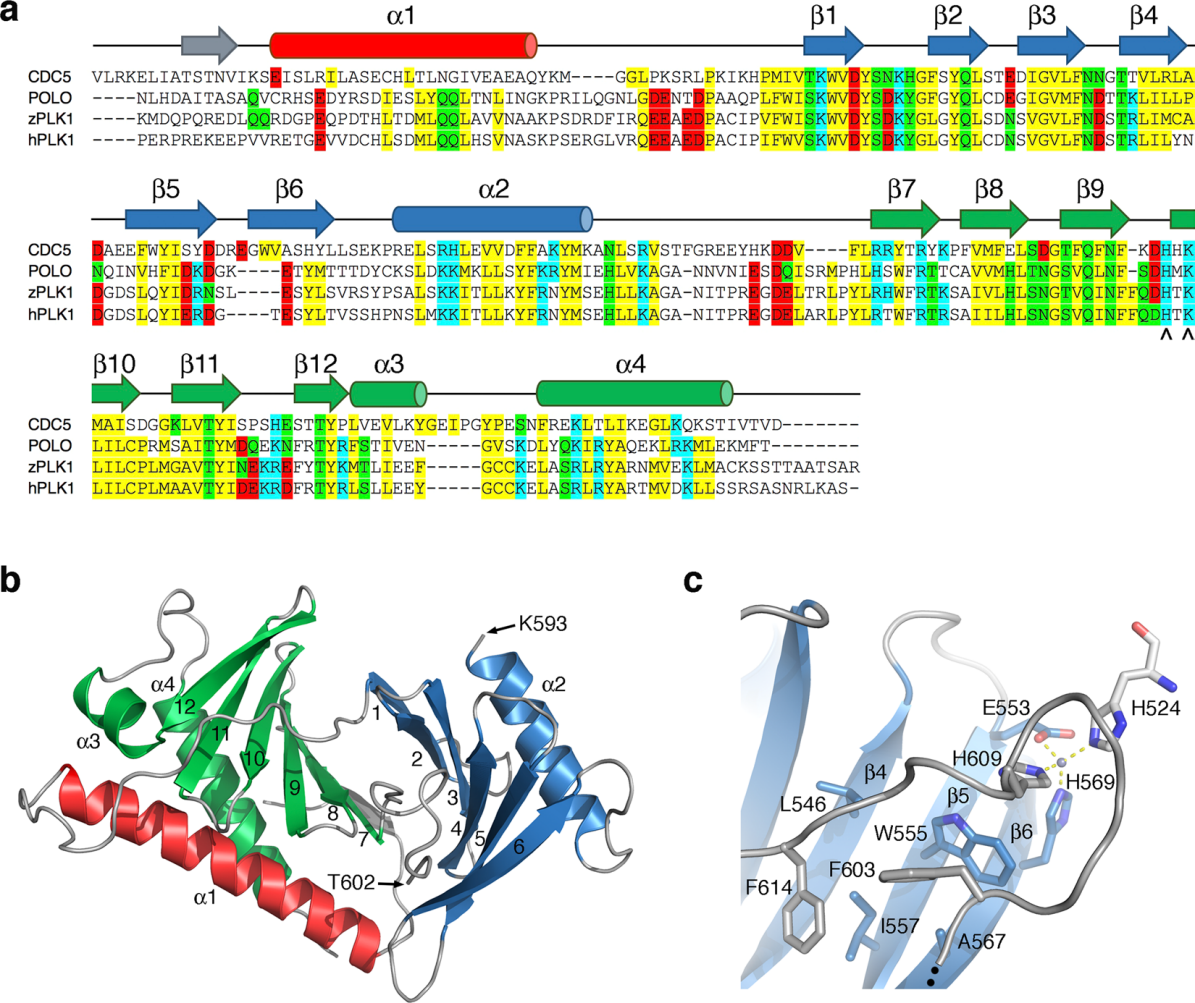
Cdc5 architecture and structure of its polo-box domain. **(a)** Sequence alignment of budding yeast Cdc5 (residues 451–705) and the drosophila (POLO, residues 333–576), zebrafish (zPLK1, residues 343–595) and human (hPLK1, residues 352–603) Plk1 homologs. Secondary structure elements are shown above the alignment and color red (polo cap), blue (first polo-box) and green (second polo box). Conserved hydrophobic (yellow), polar (green), positive charged (blue) and negative charged (red) residues are highlighted. Conserved residues involved in phosphopeptide binding are marked with (^). **(b)** Ribbon diagram of the structure of Cdc5 color coded as in **(a)**. **(c)** Detail of the interactions between the ordered region of the α2-β7 loop (residues Thr602-Phe614) and strands β5 and β6 from the first polo-box. The Zn^2+^ metal ion is shown as a grey sphere with hydrogen bonds drawn as dashed lines.

We determined the crystal structure of the polo-box domain of Cdc5 using a fragment of Cdc5 that included residues 418–705 (Fig. 1a and Table 1). Similar to other previously determined structures of Plk1, the extension preceding the polo-box domain (residues 418–459) was disordered in the structure, while residues Leu460-Asp705 adopted the characteristic polo-box domain fold with the first polo-box defined by the β1-β2-β3-β4-β5-β6-α2 structure elements, and the second by the β7-β8-β9-β10-β11-β12-α3-α4 structure elements (Fig. 1b). The loop connecting the two polo boxes (Ala594-Ser601) was disordered in this structure. The distance between the last ordered residue from the first polo-box (Lys593) and the first ordered residue in the second polo-box (Thr602), as well the conformation of residues Thr602-Asp611 folding back towards β5-β6 rather than reaching across the first polo-box towards α2, suggested that the loop may had been proteolytically cleaved during crystallization (Fig. 1b–c). The side chains of Glu553, His569 and His609, as well as that of His524 from a symmetry related molecule, coordinate a zinc metal ion from the crystallization solution (Fig. 1c). This metal ion further stabilizes the conformation of the ordered portion of the loop and places the phenyl group of Phe603 inside an exposed hydrophobic pocket delimited by Leu546, Trp555, Ile557 and Ala567 (Fig. 1b and Supplementary Fig. 1a).

**Table 1:** Data collection and refinement statistics.

	Cdc5	Cdc5-Spc72	Cdc5-Dbf4
**Data Collection**			
Beamline	08B1; CLS	17-ID; APS	17-ID; APS
Wavelength (Å)	1.2834	0.9795	0.9762
Space group	P 2_1_ 2_1_ 2_1_	P 2_1_	P 6_5_
Cell dimensions: a, b, c	51.1, 65.9, 96.1	51.5, 68.2, 86.2	134.6, 134.6, 75.3
α, β, γ	90, 90, 90	90, 102.6, 90	90, 90, 120
Resolution (Å)*	48.1–1.8 (1.86–1.8)	42.1–2.7 (2.78–2.7)	50–3.5 (3.63–3.5)
Reflections (unique/total)	58,352 / 235,329	16,158 / 93,716	10,923 / 205,926
Completeness (%)	100 (99.9)	99.9 (99.4)	100 (100)
CC_1/2_ (%)	99.7 (35.8)	99 (31.7)	99.9 (36.7)
I/σ(I)	12.58 (0.96)	9.1 (1.56)	17.3 (0.8)
Redundancy	4 (3.8)	5.8 (4.9)	19.8 (20.9)
**Refinement**			
Resolution (Å)	48.1–1.8	42.1–2.7	35.8–3.6
Completeness (%)	100	99.9	99.5
R_work_/R_free_ (%)	18.1 / 19.7	21.4 / 25.7	31.4 / 32.2
**Ramachandran plot (%)**			
Favoured /Outliers	98.3 / 0	93.6 / 0.2	87.4 / 0.9
rmsd in bonds (Å)	0.004	0.004	0.003
rmsd in angles (°)	0.885	0.805	0.504

^*^Data in the highest resolution shell is shown in parentheses.

**Figure 2:**
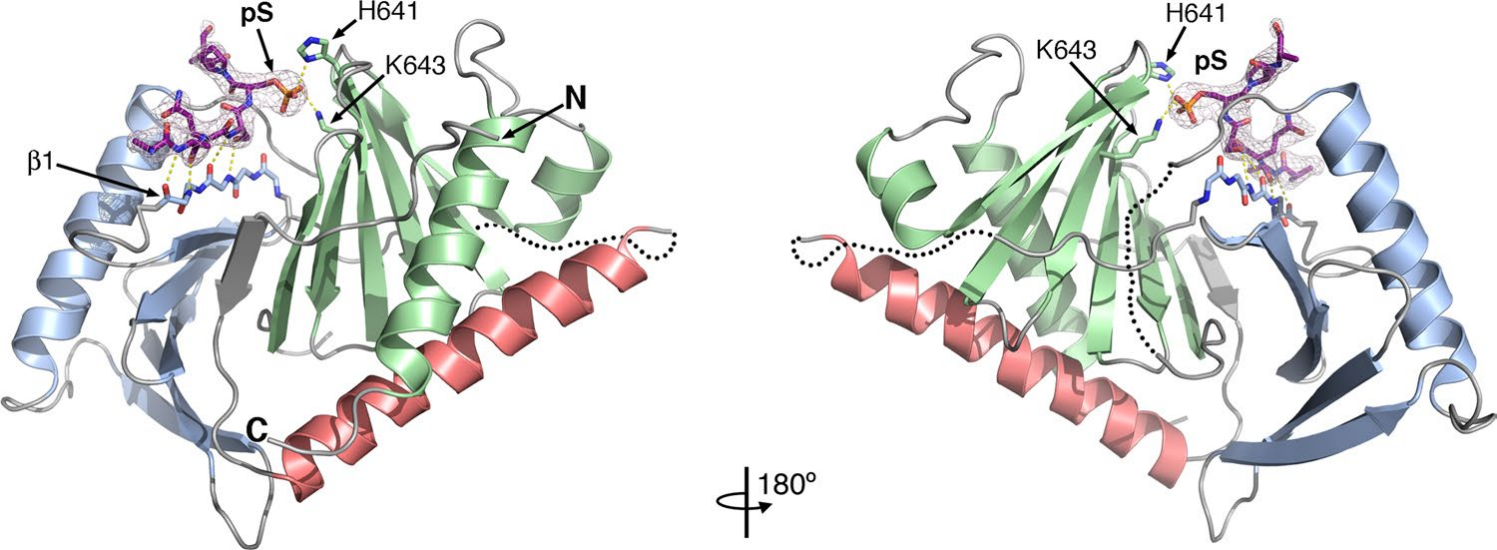
Structures of the polo-box domain (PBD) of Cdc5 bound to Spc72^P^. Opposite views of the PBD of Cdc5 bound to the Spc72^P^ peptide (magenta). Spc72^P^ binds at the groove defined by the two polo boxes forming a short antiparallel β-sheet with the β1 strand. The 2Fo-Fc electron density map around the Spc72^P^ peptide contoured at 1.2σ is shown as a grey mesh.

To confirm how Cdc5 recognizes phosphorylated substrates, we then solved the structure of Cdc5 bound to a phosphorylated peptide derived from the spindle-pole body protein Spc72 (Spc72^P^, ^227^SLA**QSpSP**AGSQ^237^). Spc72^P^ is a well-studied binding partner of Cdc5 that contains the conserved phosphorylated motif recognized by polo-box domains^34^. As expected, the Spc72^P^ phosphopeptide bound along the groove determined by the two polo-boxes and adopts an extended conformation that runs antiparallel to β1, effectively extending the β-sheet of the first polo-box by one strand (Fig. 2). The strictly conserved His641 and Lys643 cradle the phosphate group (Figs. 1a, 2), confirming that Cdc5 recognizes phosphorylated substrates the same way as other Plk1 homologs (Supplementary Fig. 2).

### Dbf4 binds a surface of the polo-box domain opposite to the pS/T-binding pocket

The ^83^RSIEGA^88^ motif, located upstream of the BRCT domain of Dbf4, is necessary for the interaction with Cdc5^23,27^. This motif lacks the X-S-p(S/T)-(P/X) sequence recognized by polo-box domains and point mutations on the pS/T-binding pocket of Cdc5 do not affect the interaction with Dbf4^27^, suggesting that Dbf4 recognizes a different surface of Cdc5.

**Figure 3:**
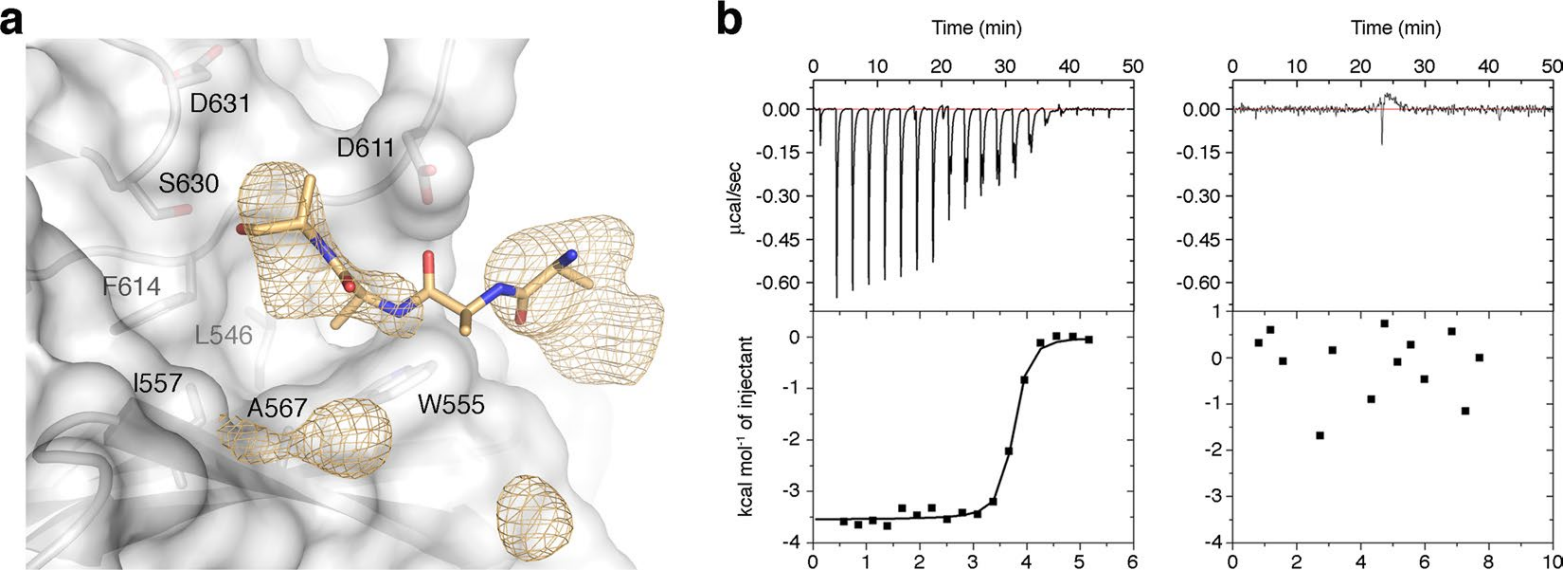
The Dbf4 peptide binds to the hydrophobic surface opposite to the pS/T-binding pocket. **(a)** Structure of the PBD of Cdc5 in the presence of the Dbf4 peptide. The Fo-Fc>0 electron density map around the hydrophobic pocket opposite to the phosphopeptide binding site is shown as a yellow mesh contoured at 2.5σ with a tetrapeptide modelled for reference (yellow sticks). **(b)** Isothermal calorimetry data and analysis for the titration of the Dbf4 into the polo-box domain of Cdc5^PBD^ (left) or Cdc5 ^PBD^-A567W (right).

To elucidate how Dbf4 interacts with Cdc5, we next solved the structure of the polo-box domain in the presence of a Dbf4-derived peptide (^76^RARIERA**RSIEGA**VQVSKGTG^96^). Crystals of this complex diffracted X-rays to lower resolution (~3.5 Å), but the structure could be readily determined by molecular replacement using the polo-box domain of the Cdc5-Spc72^P^ structure (Table 1) The polo-box domain was virtually identical to the other two structures (r.m.s. deviations of 0.86 and 0.66 Å with the apo- and Spc72^P^-bound structures, respectively). As expected, the pS/T-binding pocket was not occupied by Dbf4. However, there was not obvious electron density for the Dbf4 peptide anywhere around the polo-box domain. After a cycle of refinement, the surface opposite to the pS/T-binding pocket showed significant peaks (>2.5σ) on the Fo-Fc electron density maps (Fig. 3a). While these additional electron densities lacked connectivity and could not account for the entire Dbf4 peptide, they could accommodate few amino acids. Intriguingly, these electron density peaks were close to the hydrophobic pocket defined by residues Leu546, Trp555, Ile557 and Ala567 from Cdc5 and coincided with the position that Phe603 occupies in the structure of the polo-box domain of Cdc5 on its own (Fig. 1c).

The ^83^RSIEGA^88^ motif defines the minimal region of Dbf4 necessary for binding to Cdc5. Mutation of Ile85 within this motif completely abrogates binding to Cdc5^27^. We speculated that Ile85 could occupy the hydrophobic pocket defined by Leu546, Trp555, Ile557 and Ala567 (Fig. 3a and Supplementary Fig. 1). If this was the case, Glu86 would bind close to Asp611 and Asp631 from polo-box domain, and this could explain why a Dbf4-E86K peptide binds to the polo-box domain of Cdc5 with higher affinity than the wild-type Dbf4 peptide (Supplementary Fig. 1)^27^. To test whether this surface of the polo-box domain was important for the interaction with Dbf4, we introduced a A567W mutation on the polo-box domain to occlude the hydrophobic pocket. This variant of the polo-box domain was stable and monodisperse in solution as judged by size-exclusion chromatography and dynamic light scattering. Using isothermal calorimetry, we then compared the binding affinities of the Dbf4 peptide to the polo-box domain and the polo-box domain of Cdc5 carrying the A567W substitution. In good agreement with previously published data, the Dbf4 peptide bound to the polo-box domain of Cdc5 with a k_D_ ~ 0.5 μM (Fig. 3b and^27^). Conversely, the Dbf4 peptide failed to interact with the A567W variant of the polo-box domain (Fig. 3b), indicating that the integrity of the hydrophobic surface of the polo-box domain of Cdc5 opposite to the pS/T-binding pocket is important for Dbf4-peptide binding.

### Dbf4 and Spc72^P^ bind simultaneously and non-competitively to Cdc5

**Figure 4:**
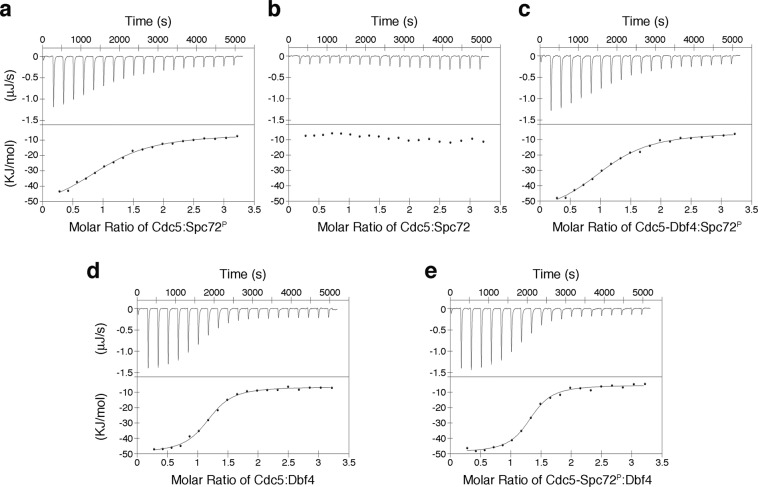
Cdc5 interacts simultaneously and non-competitively with Spc72^P^ and Dbf4. Isothermal calorimetry data and analysis for the titration of the Spc72^P^ peptide **(a)**, its non-phosphorylated version (Spc72) **(b)**, and the Dbf4 peptide **(d)** into the polo-box domain of Cdc5. **(c)** Titration of the Spc72^P^ peptide into the polo-box domain of Cdc5 pre-incubated with the Dbf4 at 1:4 molar excess. **(e)** Titration of the Dbf4 peptide into the polo-box domain of Cdc5 pre-incubated with the Spc72^P^ at 1:4 molar excess.

Since Dbf4 and Spc72^P^ bind to opposite faces of the polo-box domain, we next tested whether both peptides could simultaneously bind to Cdc5. Using isothermal calorimetry, we confirmed that the Spc72^P^ peptide interacted with the polo-box domain of Cdc5, and the interaction was phosphorylation dependent (Fig. 4a,b). The binding isotherms for the Cdc5-Dbf4 and Cdc5-Spc72^P^ complexes point to saturation occurring at around 1:3 molar ratios of Cdc5:peptide (Figs. 4a,d). Therefore, we tested whether Spc72^P^ could bind with Cdc5 that had been pre-incubated with Dbf4 at 1:4 molar excess. We found that Spc72^P^ bound to Cdc5 and Cdc5-Dbf4 with similar affinities (Figs. 4a,c). The reciprocal experiment yielded similar results (Fig. 4d,e), confirming that Cdc5 can bind both substrates simultaneously *in vitro*.

**Figure 5:**
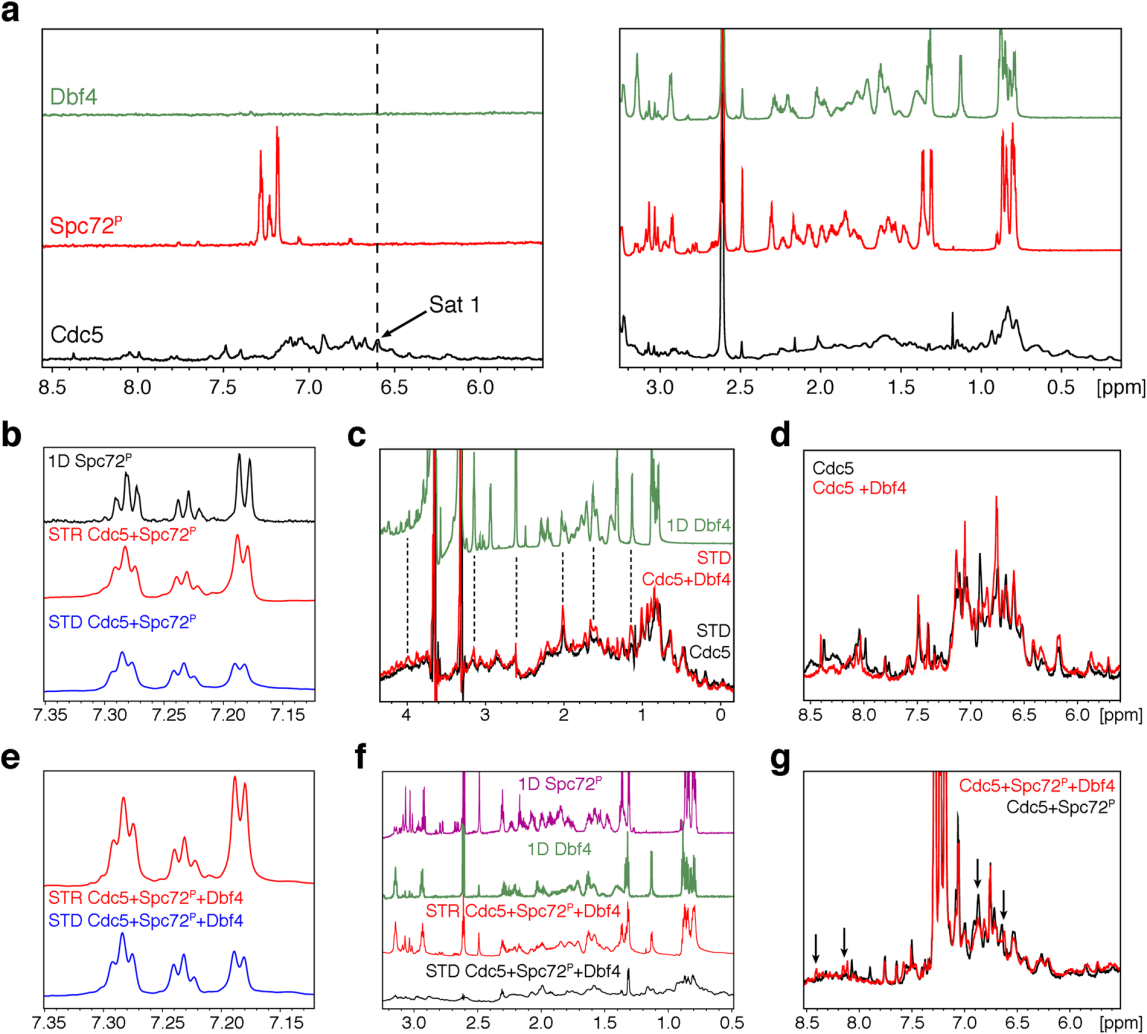
NMR spectra probing the Cdc5-substrate interactions. **(a)** One dimensional ^1^H NMR spectra for Cdc5, Spc72^P^, and Dbf4. The Cdc5-selective saturation frequency used in the STD experiments is indicated. **(b)** Aromatic expansion of the STD NMR spectrum of Scp72^P^ acquired in the presence of Cdc5. The 1D ^1^H and STD reference (STR) spectra of Scp72^P^ in the absence and presence of Cdc5 are shown for comparison. **(c)** Overlay of the Cdc5 and Cdc5-Dbf4 STD spectra. Dashed lines highlight Cdc5-Dbf4 peaks with increased intensities that align with free Dbf4 (green) chemical shifts. **(d)** Overlay of the aromatic region of 1D ^1^H NMR spectra for Cdc5 and Cdc5-Dbf4. **(e)** Aromatic expansion for the STD and STR spectra of the Cdc5-Scp72^P^-Dbf4 complex. **(f)** Aliphatic region of the Cdc5-Spc72^P^-Dbf4 STD spectra. 1D ^1^H NMR spectra for each peptide and the STR spectrum of the Cdc5-Spc72^P^- Dbf4 complex are shown for reference. **(g)** Overlay of the aromatic region of 1D proton spectra for the Cdc5-Spc72^P^ and Cdc5-Scp72^P^-Dbf4 complexes. Arrows indicate chemical shift changes caused by Dbf4 binding.

Although measurements at different excess molar ratios yielded consistent results, we could not exclude the possibility that the pre-bound peptide was displaced by the titrating peptide. To test whether both peptides were bound simultaneously to the polo-box domain, we used saturation-transfer difference (STD) NMR. One dimensional ^1^H NMR spectra were acquired for Cdc5, Dbf4 and Spc72^P^ to determine suitable saturation frequencies selective for Cdc5 (Fig. 5a). Since there was significant spectral overlap between the aliphatic region of Cdc5 and the two peptides, we selected a saturation frequency within the aromatic region of Cdc5 (6.604 ppm). The Dbf4 peptide lacks aromatic residues and had no signals in this region, thereby avoiding the risk of non-selective saturation. This was confirmed by acquiring control STD experiments in the absence of Cdc5, which resulted in no observable STD signals (Supplementary Fig. 3). Spc72^P^ had a minor peak at around 6.7 ppm and the STD spectra acquired in the absence of Cdc5 resulted in additional Spc72^P^ resonances (Supplementary Fig. 3). To account for this saturation leak through, STD spectra of Spc72^P^ observed in the presence of Cdc5 were compared to those in its absence.

We then acquired STD experiments for the Cdc5-Dbf4 and Cdc5-Spc72^P^ complexes. The saturation transfer for Spc72^P^ resulted in a signal significantly greater than the phosphorylated peptide alone (Fig. 5b, Supplementary Table 1). The results for the Dbf4 peptide were less clear due to signal broadening and spectral overlap with Cdc5 (Fig. 5c). However, comparing the STD spectra acquired in the absence and presence of Dbf4, we were able to identify several peaks with increased intensities that were consistent with the positions of Dbf4 resonances in the absence of Cdc5. The interaction between Cdc5 and Dbf4 was also evident from the chemical shift changes in the aromatic resonances of Cdc5 upon addition of Dbf4 (Fig. 5d). Since Dbf4 has no resonances in this region, the spectral differences could only be explained due to Cdc5 chemical shift changes caused by the binding of Dbf4.

When analyzing the ternary complex (Dbf4-Cdc5-Spc72^P^), we observed STD signals for Spc72^P^ (Fig. 5e). The normalized STD intensities (i.e. the STD/STR ratios) indicated that the binding affinity was similar to that of the Cdc5-Spc72^P^ complex (Supplementary Table 1). As for Dbf4, the STD detection was impaired by spectral crowding and weak intensities, similar to the Cdc5-Dbf4 complex (Fig. 5f). However, chemical shift changes between the Cdc5-Spc72^P^ and Dbf4-Cdc5-Spc72^P^ complexes confirm that Dbf4 interacts with Cdc5 in the presence of Spc72^P^ (Fig. 5g). Overall, the STD/STR spectra confirmed that Cdc5 can bind the Spc72^P^ phosphopeptide and Dbf4 simultaneously, and that binding to one peptide does not significantly affect binding of the other.

### Targeted mutations in Cdc5 generate conditional lethal alleles *in vivo*

We have previously shown that mutations affecting the phosphopeptide-binding activity of the polo-box domain of Cdc5 are tolerated in yeast as they generate viable alleles that are not associated with strong proliferation defects^35^. However, these alleles show a striking misregulation of cell cycle checkpoints and are defective in their maintenance of genome stability^35,36^. In light of these observations, we wanted to assess the physiological impact of mutations affecting the hydrophobic pocket of the polo-box.

**Figure 6:**
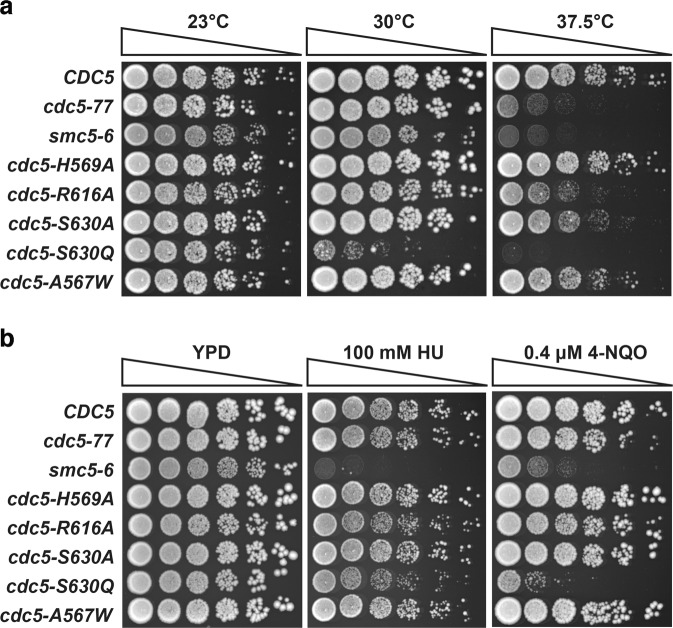
Disruption of Cdc5 hydrophobic pocket generates conditional lethal mutants. Exponential culture of the indicated mutant and control cells were spotted on solid medium as fivefold dilution series. Cells were allowed to grow in temperature-controlled incubators for 36–72 hours until individual colonies were visible. **(a)** Effect of temperature on the proliferation of *cdc5* mutants. Cells were incubated at 23 °C, 30 °C, and 37.5 °C on solid YPAD medium. **(b)** Sensitivity of *cdc5* mutants to DNA replication inhibitor and DNA damaging agent. Cells were grown at 23 °C, and mutants defective in DNA repair (*smc5-6*^37^) and Cdc5 kinase activity (*cdc5-77*^35^) were included as controls. (n = 5).

We introduced point mutations at residues delimiting the surface where Dbf4 binds within one copy of the *CDC5* gene in diploid yeast, and the resulting heterozygous mutant strains were sporulated. After dissection of sporulated tetrads, all haploid segregants carrying *cdc5* mutations were viable and germinated normally. Interestingly, several hydrophobic pocket mutants showed reduced cell proliferation capacity at high temperature and in medium containing DNA damaging agents (Fig. 6). In particular, the *cdc5-S630Q* mutant showed a striking growth defect both at high temperatures (Fig. 6a) and in the presence of 4-nitroquinoline 1-oxide (4-NQO; Fig. 6b), with very little or no growth observed under these conditions. The 4-NQO hypersensitivity of the *cdc5-S630Q* mutant was comparable to that of cells defective in the Smc5-6 complex, a known DNA repair enzyme (Fig. 6b)^37^. Importantly, the proliferation of the *cdc5-S630Q* mutant was unaffected relative to wild-type controls at the permissive temperature of 23 °C, indicating that it is a *bona fide* conditional-lethal mutant *in vivo*. Ser630 is located at the end of the β8 strand and it is held in place by hydrogen bonds with Phe614 leading to β7. Ser630 defines the back wall of the Dbf4-binding pocket (Supplementary Fig. 1c). A point mutation introducing a larger side chain (S630Q) at this position would reduce the size of the hydrophobic pocket and, thus, it would be more deleterious than one introducing a smaller side-chain (S630A). Similarly, other interactions mediated by this surface of Cdc5 would also be affected by reducing the size of the pocket.

### The *cdc5-S630Q* mutant fails to complete the segregation of its chromosomes and arrests in anaphase

**Figure 7:**
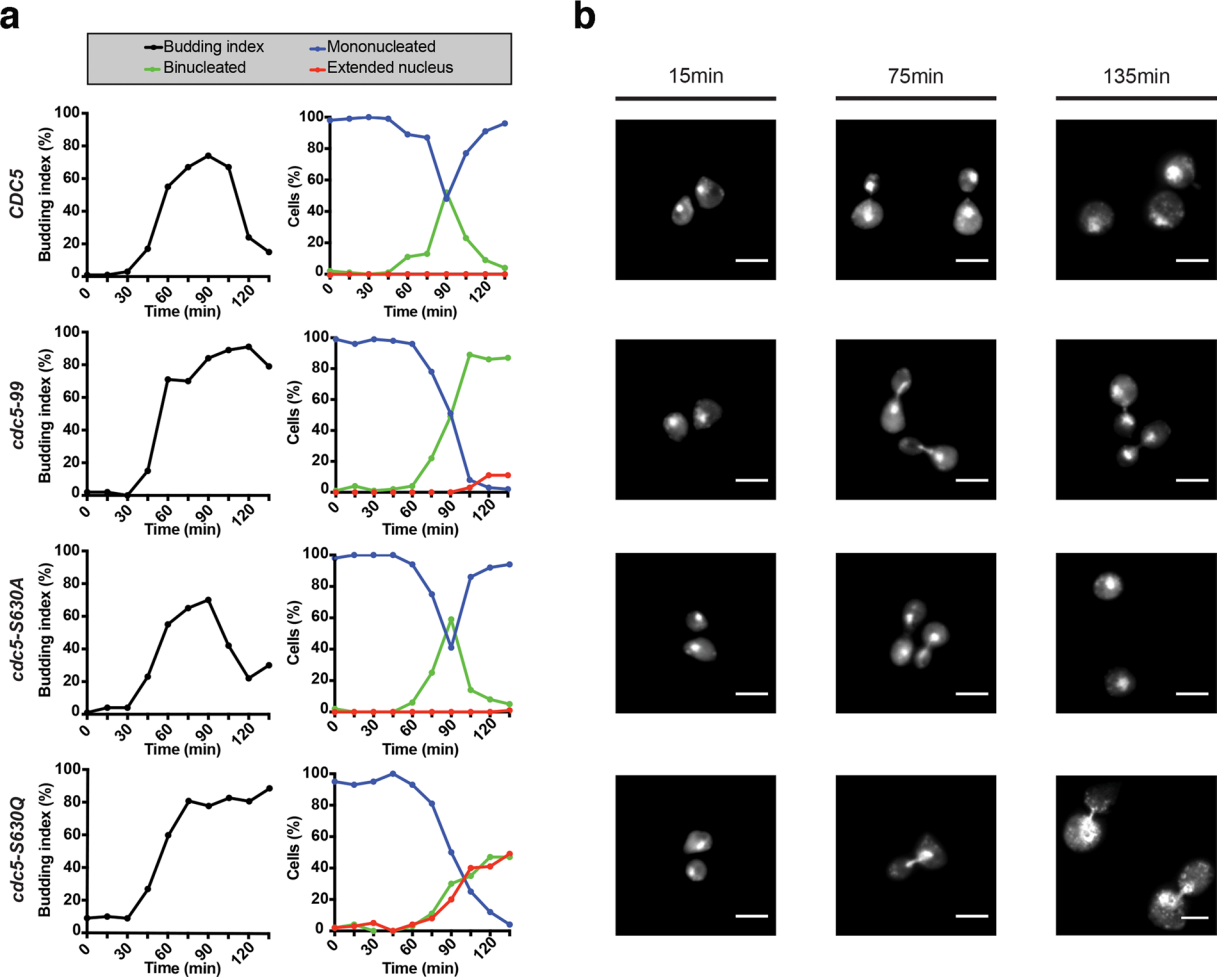
Chromosome segregation defect in *cdc5-S630Q* mutants. Exponential cultures of *cdc5-S630Q* mutant and control cells were synchronized in G1 at 23 °C using α-factor. After synchronous release of cells in fresh YPAD medium at 37 °C, samples of culture were collected at regular intervals and processed to monitor the appearance of cell cycle landmarks. (**a)** Kinetics of bud formation and nucleus separation during the cell cycle. 100 cells were counted at each time point. (**b)** Micrographs showing representative nucleus morphology at the indicated times during the time-course experiment. White bar is 5 µm. Note that the 135 min micrograph for the *cdc5-S630Q* mutant shows a slightly larger surface area than other micrographs to allow visualization of 2 cells, hence the slightly shorter scale bar. (n = 4).

Since Cdc5 function is essential for mitotic exit^35^, we hypothesized that the conditional-lethal behavior of the *cdc5-S630Q* mutant might reflect a defect in mitotic progression. To test this hypothesis, we monitored cell cycle kinetics in synchronized populations of cells. Yeast strains were first arrested in G1 phase with α-factor at 23 °C and subsequently released from this arrest into a synchronous cell cycle at restrictive temperature for *cdc5-S630Q* and *cdc5-99* mutants. The latter mutant shows the typical mitotic exit defect associated with Cdc5 inactivation and was used as a positive control in the experiment^7^. Samples of cells were taken at regular intervals during the time-course experiment to monitor the appearance of cellular landmarks by microscopy. The initial formation of buds on mother cells, which is concomitant with the early stages of DNA replication, followed similar kinetics in all strains (Fig. 7a). Whereas wild-type cells and a control mutant (*cdc5-S630A*) completed mitosis and returned to G1 normally, as evidenced by the formation of large-budded cells and their separation late in the time-course, both *cdc5-S630Q* and *cdc5-99* mutants accumulated large budded cells and failed to divide into two separate cells, even at the last time-point of the experiment (135 min; Fig. 7a). This phenotype is consistent with a late mitotic defect in *cdc5-S630Q* cells.

To more precisely pinpoint at which stage of mitosis the *cdc5-S630Q* mutant is defective, we monitored the separation of DAPI-stained nuclei in dividing cells. A synchronous time-course experiment was conducted as described above and the morphology of nuclei in mutant and control cells was classified according to three different categories reflecting the normal timeline of chromosome segregation in yeast: mononucleated, extended/irregular nucleus, and binucleated. *cdc5* mutants often show a minor delay in chromosome segregation during anaphase^8^ and typically experience a terminal arrest in late telophase (*i.e*., as binucleated cells that sometimes carry thin anaphase bridges between separated nuclei^35^). The negative (*CDC5* and *cdc5-S630A*) and positive (*cdc5-99*) control strains showed the expected behavior in their kinetics of nucleus segregation in the time-course experiment (Fig. 7a). Namely, segregation started 70 min after the release from the α-factor block for all strains and reached a maximum at 90 min post-release for *CDC5* and *cdc5-S630A* strains, whereas the *cdc5-99* mutant accumulated as a pure population of binucleated cells due to its defect in mitotic exit. In striking contrast, chromosome segregation was strongly delayed in the *cdc5-S630Q* mutant, and about 50% of the cells completely failed to segregate their DNA by the end of the experiment (see “extended nucleus” category in Fig. 7a). Examination of nucleus morphology in *cdc5-S630Q* mutants revealed cells blocked early or mid-way in the process of chromosome segregation (Fig. 7b). This phenotype was observed using a generic dye to label genomic DNA (DAPI, Fig. 7b), as well as a fluorescent marker for chromatin (histone H2A fused to mCherry, Supplementary Fig. 4a,b). Consistent with this observation, the distance that separates spindle pole bodies (SPBs), which reflects nuclear spindle length^38^, was remarkably shorter in *cdc5-S630Q* mutants relative to wild-type cells in early anaphase (*i.e*., 2.67 ± 0.35 µm *vs* 4.51 ± 0.04 µm; Supplementary Fig. 4c). An early anaphase arrest phenotype has not been observed previously in other *cdc5* mutants and likely reflects the unique nature of the molecular defect in the *cdc5-S630Q* allele. The *cdc5-S630A* mutant did not show detectable defects in chromosome segregation, indicating that the phenotype of the *cdc5-S630Q* mutant is specific. Taken together, these results indicate that the integrity of the hydrophobic surface of the polo-box domain opposite to the pS/T-binding pocket is important for effective segregation of chromosomes in anaphase.

## Discussion

Genetic and structural studies have described the hierarchy of interactions involving the polo-box domain of Plks, as well as the molecular determinants mediating recognition of phosphorylated targets^31^. Phosphorylation-independent interactions mediated by polo-box domains are also common but have received less attention^23,27,32^. Here we demonstrate that the polo-box domain of Cdc5 uses distinct surfaces to bind phosphorylated and non-phosphorylated substrates. *In vitro*, Dbf4 and phosphorylated peptides can bind simultaneously and not competitively to the polo-box domain of Cdc5. Dbf4-binding to Cdc5 during the spindle positioning checkpoint promotes DDK-mediated phosphorylation of the polo-box domain of Cdc5, and this presumably prevents Cdc5 from recognizing its phosphorylated binding substrates in the MEN pathway^23^. The structure of Cdc5 bound to a Dbf4 peptide cannot recapitulate the spatial organization of the complex formed by Cdc5 and DDK, but it is plausible that additional regions of Dbf4 or Cdc7 (the kinase subunit of the DDK complex) beyond the interacting motif partially block Cdc5 access to its phosphorylated substrates.

**Figure 8:**
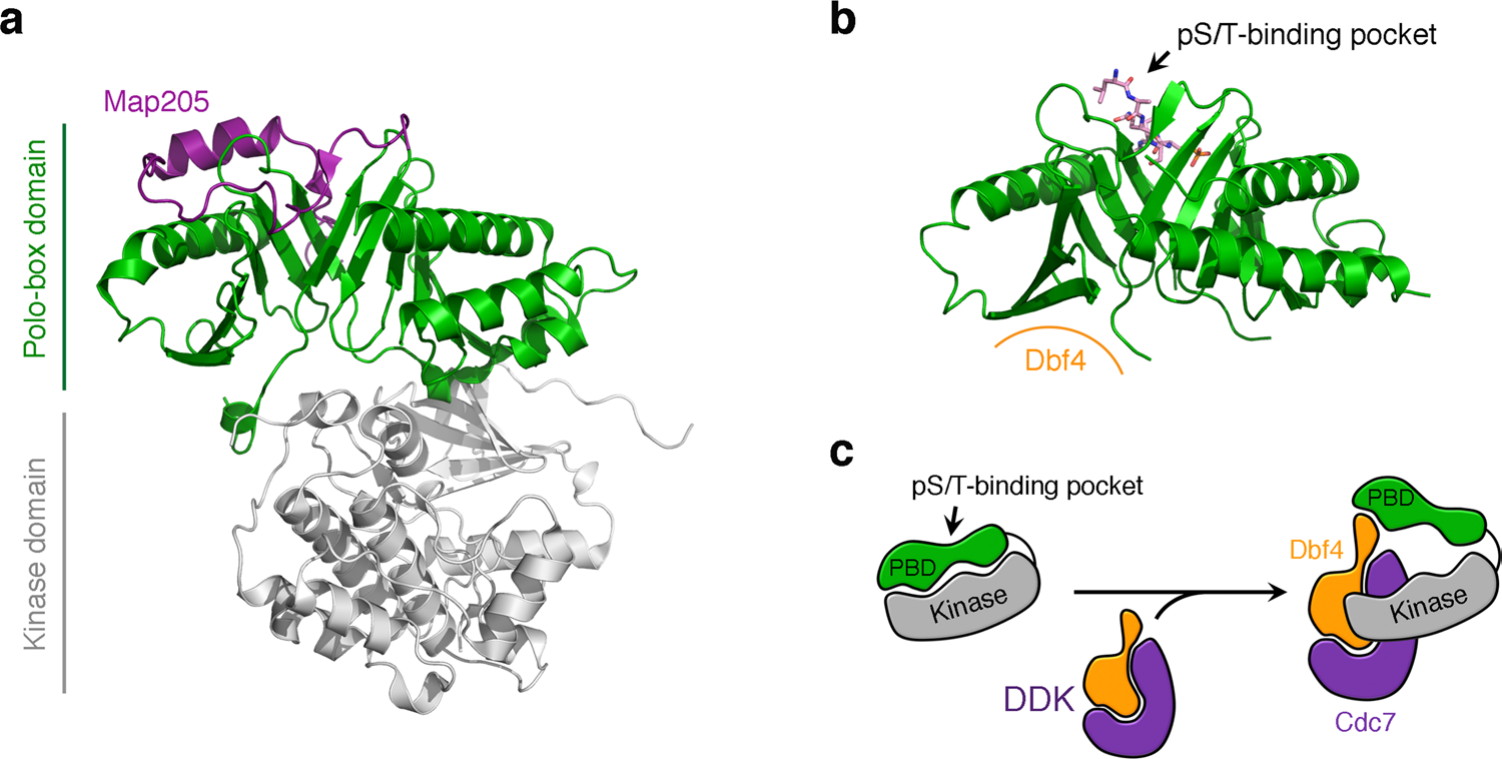
Comparison of yeast Cdc5 and zebrafish Plk1. **(a)** Ribbon diagram of the structure of zebrafish Plk1 in complex with Map205 (purple). The kinase domain (grey) and the polo-box domain (green) are labeled. **(b)** Ribbon diagram of the structure of the polo-box domain of Cdc5 in complex with the Spc72^P^ peptide shown in the same orientation as **(a)**. The Spc72^P^ is shown as color-coded sticks and the Dbf4-binding interface indicated in orange. **(c)** Model depicting how the DDK complex may bind to Cdc5. Dbf4 interacts with the polo-box domain (PBD) of Cdc5, but additional interactions mediated by the Dbf4 or Cdc7 subunits of the DDK complex may contribute to the interaction and potentially alter the kinase activity of Cdc5.

The Dbf4-Cdc5 interaction is also necessary to phosphorylate the Mms4-Mus81 nuclease^29^. If Dbf4-binding to Cdc5 enhances its kinase activity, it would explain how the Dbf4-Cdc5 interaction promotes Cdc7 and Cdc5 phosphorylation of Mms4-Mus81. The crystal structure of zebrafish Plk1 (zPlk1) is the only one containing both the kinase and polo-box domains (Fig. 8a). Association of the kinase and polo-box domains is responsible for the auto-inhibition of the kinase activity of zebrafish^33^. It has been proposed that binding of phosphorylated substrates loosens the interaction, in turn releasing the inhibition^30,33,39,40^. Indeed, the kinase domain of Plk1 has higher kinase activity than the full-length protein. Although Cdc5 auto-inhibition has not been studied, the interface mediating the interaction between the polo-box and kinase domains is conserved. Therefore, the compact form visualized in the structure of zPlk1 likely provides a general description for the auto-inhibited state of Plks. Superimposition of the polo-box domain of Cdc5 onto that of zPlk1 reveals that the Dbf4-binding surface overlaps with the surface where the kinase domain binds (Fig. 8a,b). Although only the RSIEGA motif of Dbf4 is required for the interaction with the polo-box of Cdc5, binding of the DDK complex likely introduces additional contacts that may detach the polo-box domain from the kinase domain of Cdc5 (Fig. 8c).

The Cdc5-Dbf4 structure is the first of a polo-box domain in complex with a binding partner that interacts through a surface beyond the conserved pS/T-binding pocket and, thus, provides new ways of modulating Plk activity. Importantly, the interaction between Dbf4 and Cdc5 has allowed us to identify a conserved residue (Ser630) that is critical for Cdc5 function. Mutation of this residue results in an unexpected defect in anaphase progression *in vivo*. Classic studies by Hartwell and colleagues have shown that *cdc5* mutants arrest in the cell cycle with fully segregated chromosomes in a telophase-like terminal state^41,42^. This phenotype reflects the mitotic exit defect of *cdc5* mutants, and more recent analyses of Cdc5-deficient cells have revealed that they are also defective in pre-telophase events, such as the release of Cdc14 from the nucleolus in early anaphase^43^ and the phosphorylation of cohesin at the metaphase-anaphase transition^8^. Importantly, these defects were not previously associated with severe consequences on chromosome segregation, as we have observed in *cdc5-S630Q* mutant cells. This strongly suggests that the novel Dbf4-binding pocket we have characterized is promoting a *hitherto* unknown function of Cdc5 at the metaphase-anaphase transition and/or in early anaphase.

Although possible, it seems unlikely this function is related to Cdc5 phosphorylation of Mcd1/Scc1, since complete abrogation of this regulatory event results in a mild delay in cohesin inactivation and does not lead to a strong anaphase arrest phenotype^8^. Rather, we favor the hypothesis that the polo-box domain of Cdc5 controls a currently unknown but essential process in mid-mitosis. This interpretation is consistent with our previous observation that inactivation of the canonical phosphopeptide-binding activity of Cdc5 PBD does not impact cell viability, whereas full inactivation/deletion of the polo-box domain is a lethal event *in vivo*^35^. It is also interesting to note that cells defective in Cdc7, the main binding partner of Dbf4, were originally characterized as defective in the medial stage of cell division^42^, similar to the phenotype we observed in *cdc5-S630Q* mutants. It is thus possible that the regulatory interplay between Cdc5 and Dbf4 goes beyond these two proteins and requires the Cdc7 kinase to take effect. Indeed, the Dbf4 peptide does not affect the kinase activity of Cdc5 (Supplementary Fig. 5), reinforcing the idea that additional interactions may be required. Apart from this scenario, it is important to consider the fact that the Dbf4-binding pocket on Cdc5 might mediate interactions with other binding partners, including the Cdc5 kinase domain itself, thus leading to a complex network of combinatorial interactions for the regulation of mitosis by Cdc5/PLKs. It will be interesting to test these possibilities and their physiological implications in future studies.

## Methods

### Production of the Cdc5 polo-box domain

The plasmid encoding *Saccharomyces cerevisiae* Cdc5 (pMW537) was a kind gift from Prof. Michael Weinreich. For the crystallographic analysis, we subcloned the polo-box domain of Cdc5 (residues 418–705) in the pPROEX HTa vector (pAG 8531) (Invitrogen Life Technologies). The Cdc5-A567W variant of the polo-box domain was generated by site-directed mutagenesis and clones were confirmed by DNA sequencing (Nanuq, McGill University and Génome Québec Innovation Centre). Expression and solubility of the Cdc5 polo-box domain was optimized as described earlier^44^. For large scale expression, Cdc5 plasmids were transformed in BL21 (DE3) cells containing a plasmid encoding for rare tRNAs. Cultures were grown in Luria-Bertani media to OD_600_ ~ 0.6, induced by addition of 2 mM isopropyl ß-D-1-thiogalactopyranoside, and incubated overnight at 16 °C with orbital agitation.

Cell pellets were resuspended in 50 mM TRIS-HCl pH 7.0, 500 mM NaCl, 1.4 mM 2-mercaptoethanol, 5% glycerol and lysed by sonication. Lysates were cleared by centrifugation at 39,000 *g*, and the supernatants loaded onto a HiTrap nickel-chelating HP column (GE Healthcare). His_6_-tagged Cdc5 was eluted at 240 mM imidazole. The histidine tag was removed with tobacco etch virus (TEV) protease, and tagless Cdc5 further purified by affinity and size-exclusion (Superdex 75 (10/300) GL, GE Healthcare) chromatography. Purified Cdc5 was kept in storage buffer (50 mM TRIS-HCl pH 7.0, 200 mM NaCl, 1.4 mM 2-mercaptoethanol, 5% glycerol). Protein concentration was determined using the Beer-Lambert equation with an extinction coefficient of 38,850 M^−1^cm^−1^ for Cdc5, and 44,350 M^−1^cm^−1^ for Cdc5-A567W. Purified proteins were monodisperse in solution as judged by dynamic light scattering.

For the circular dichroism measurements, Cdc5 and Cdc5-A567W (7 µM) were buffer exchanged to 50 mM TRIS-HCl pH 7.0, 150 mM NaF, 1.4 mM 2-mercaptoethanol, 5% glycerol. Data was collected at room temperature using a Chirascan instrument from Applied Photophysics. Twenty iterations were done for each run, and measurements were recorded between 190–250 nm wavelength. Data was analyzed using the software provided with the Chirascan system.

### Crystallization and structure determination

Crystals of Cdc5 grew in 100 mM Bis-TRIS Propane pH 8.5, 20% PEG 3350 (v/v), 200 mM sodium formate, and cryo-protected with 8% glycerol. A complete data set was collected at the 08B1 beam line at the Canadian Light Source, and data was processed and scaled using XDS^45^ (Table 1). The Cdc5 structure was determined by molecular replacement using a fragment of the polo-box domain of human Plk1 (PDB ID: 1Q4O) as the search model. An initial model was built using auto-build in PHENIX and improved by iterative cycles of manual model building in Coot and refinement in PHENIX^46,47^ (Table 1).

Peptides derived from Spc72 (^227^SLAQSpSPAGSQ^237^) and the polo-interacting region of Dbf4 (^76^RARIERARSIEGAVQVSKGTG^96^) were purchased from GenScript and resuspended in storage buffer. The polo-box domain of Cdc5 (5 mg/mL) was mixed with Spc72^P^ at a 1:15 (protein:peptide) molar ratio and incubated overnight at 4 °C prior to crystallization trials. Crystals of Cdc5-Spc72^P^ grew in 100 mM Bis-TRIS pH 6.5, 25% PEG3350 (v/v), 200 mM MgCl_2_, 100 mM CsCl, and were cryoprotected with 10% glycerol. Cdc5 (5 mg/mL) was mixed with Dbf4 at a 1:10 (protein:peptide) molar ratio and incubated overnight at 4 °C prior to crystallization trials. Crystals of Cdc5-Dbf4 grew in 20% PEG 3350, 200 mM sodium potassium tartrate, 10 mM trimethylamine hydrochloride, and were cryo-protected with 14% glycerol. Complete data sets of the Cdc5-Spc72^P^ and Cdc5-Dbf4 complexes were collected at the 17-ID beam line at Advanced Photon Source (at Argonne National Laboratory), and processed using MOSFLM^48^ (Table 1). The structures were determined by molecular replacement using the polo-box domain of Cdc5 as the search model. Initial models of the Cdc5-Spc72^P^ and Cdc5-Dbf4 structures were further improved by iterative cycles of manual model building in Coot and refinement in PHENIX^46,47^ (Table 1). Figures showing molecular structures were generated using PyMOL^49^. Crystal structures have been deposited in the Protein Data Bank, www.rcsb.org (PDB ID: 6MF4, 6MF5, and 6MF6).

### Isothermal calorimetry experiments

The Cdc5-Spc72^P^ and Cdc5-Dbf4 interactions were analyzed using the Nano ITC instrument (TA Instruments), while the interaction between Cdc5-A567W and Dbf4 was analyzed using the MicroCaliTC_200_ instrument (Malvern Instruments Inc.). Peptides derived from Dbf4 (^73^EKKRARIERARSIEGAVQVSKGTG^96^), and Spc72^P^ (^222^DKEEFLSLAQSpSPAGSQ^237^), as well as a non-phosphorylated variant of the Spc72 peptide were purchased from GenScript and resuspended in storage buffer supplemented with 20 mM EDTA pH 8.0. The Cdc5-peptide interactions analyzed using the Nano ITC instrument were designed by filling the chamber cell with 30 µM protein and the injection syringe with 280 µM peptide. The ternary complex (Dbf4-Cdc5-Spc72^P^) was analyzed by filling the chamber cell with either Cdc5-Dbf4 or Cdc5-Spc72^P^ (at a 1:4 molar ratio of protein:peptide), and the injection syringe with 280 µM peptide. Data was processed using the NanoAnalyze program (TA Instruments). The interaction between Cdc5-A567W and Dbf4 was analyzed with 45 µM protein in the chamber cell and 1.2 mM peptide in the injection syringe. Data was processed using the MicroCaliTC_200_ program (Malvern Instruments Inc.).

### Saturation Transfer Difference (STD) Analysis

All samples were either dissolved or buffer exchanged into 20 mM sodium phosphate buffer pH 7.0, 200 mM NaCl, 20 mM EDTA pH 8.0, 1.4 mM 2-mercaptoethanol, >99% D_2_O. The concentration of Cdc5 was kept at 20 μM, while the Dbf4 (^73^EKKRARIERARSIEGAVQVSKGTG^96^) and Spc72^P^ (^222^DKEEFLSLAQSpSPAGSQ^237^) peptides were at 500 μM, unless otherwise specified. STD experiments were acquired at 298 K on a Bruker Avance 850 MHz spectrometer with a TXI probe^50–52^. The spectra were acquired with 32 K points, a spectral width of 16 ppm and a carrier frequency of 4.7 ppm. STD NMR spectra were acquired with 2 K scans, while saturation transfer reference (STR) spectra were acquired with 256 scans. Spectra were processed in Topspin 3.5 using an exponential multiplication window function with a 3 Hz line broadening. STD/STR ratios were then compiled for the Spc72^P^ peptide in the absence and presence of Cdc5 or Cdc5 bound to Dbf4.

### Yeast strains

All yeast strains used in this study were constructed in the W303 background and their relevant genotypes are summarized in (Supplemental Table 2). Standard conditions and procedures were used for yeast growth, sporulation and tetrad dissection. Growth of temperature-sensitive (ts) mutants in liquid media was performed at 23 °C (permissive temperature) and 37 °C (non-permissive temperature).

### Construction of *cdc5* mutant strains

Site-directed mutagenesis of plasmid p409 (YCplac22-*CDC5*-*T*_*ADH1*_-*HIS3MX6*) was used to insert mutations in *CDC5* gene^35^. Mutant alleles were inserted at their endogenous locus by plasmid digestion and subsequent transformation into a diploid wild-type (WT) strain. Plasmid digestion generated a DNA fragment encompassing *CDC5* ORF along with its *HIS3MX6* marker. The backbone of p409 was lost during the yeast transformation and integration of selected mutations was confirmed through sequencing of the *CDC5* locus. Diploid transformants were then sporulated and dissected. Haploid segregants were isolated and the presence of the required mutation (and absence of any secondary mutation) was established by whole gene sequencing.

### Proliferation assays

The temperature-sensitive (ts) growth behavior of *cdc5* mutants was monitored on solid YPAD medium at the following temperatures: 23, 30 and 37.5 °C. Their sensitivity to DNA replication inhibitors and DNA damaging agents was assessed at 23 °C on YPAD medium containing either 100 mM hydroxyurea (HU) or 0.4 μM 4-nitroquinoline N-oxide (4-NQO). In all cases, cultures of yeast strains were diluted fivefold and subsequently spotted on solid medium, using a starting OD_600_ of 0.2 in the first dilution^36^. Yeast were allowed to grow for approximately 36–72 hours at the indicated temperatures prior to scanning plates. The experiment was repeated 5 times (n = 5).

### Budding index assay

Conditions for growth and cell synchronization/release are as described earlier^53^. To compare strains that arrest in mitosis with those that do not, α-factor was re-added to all cultures 75 minutes after the initial G1 release. This was necessary to prevent yeast strains from re-starting a new cell cycle after completion of the first cycle. After the initial α-factor release, 1 mL aliquots of yeast cultures were taken every 15 minutes, for a total of 135 minutes. Samples of cells were centrifuged and then suspended in 1 mL of a 70% ethanol solution for 12–24 hours. Cells were subsequently resuspended in 500 µL 0.1 M potassium phosphate buffer pH 6.4 and briefly sonicated before performing bud morphology assessment by light microscopy, as previously published^53^. This experiment was repeated 4 times (n = 4).

### Fluorescence microscopy

Visualization of DNA morphology was performed on a Nikon Eclipse T*i*2 inverted microscope. The microscope was equipped with a 100×/NA 1.45 objective. Staining of the nuclei was performed with 4′,6-diamidino-2-phenylindole (DAPI) at a final concentration of 2 µg/ml in cells suspended in 0.1 M potassium phosphate pH 6.4 buffer. Aliquots of cells containing DAPI were incubated at room temperature (~22 °C) in the dark, for a total duration of 20 minutes before being washed with a 0.1 M potassium phosphate solution at pH 6.4. Final samples were resuspended in 100 µl 0.1 M potassium phosphate buffer pH 6.4 and 5 µl of each sample were used for observation. The criteria used to classify yeast nucleus morphology were as follow: (1) mononucleated, (2) binucleated with or without minor anaphase bridges, and (3) extended/undivided nucleus. To be classified in category 2, nuclei must be free of anaphase bridges or be connected by a small bridge only (*i.e*., the width of the bridge being smaller than the radius of the nucleus). 100 cells were scored at each time point. Pictures were taken 15, 75, and 135 min after α-factor release. This experiment was repeated 4 times (n = 4). Imaging of Spc42-GFP position in cells and calculation of distances that separate Spc42-GFP foci was performed as previously described^35,38^ using the 3D measurement module of the NIS-Elements software (Nikon Instruments Inc.). This experiment was repeated 3 times (n = 3).

### *In vitro* kinase assays

Full-length Cdc5 was produced and purified as previously described^7^. The *in vitro* kinase assays were done as described earlier^35^ with minor modifications. In brief, 0.125 pmol of Cdc5 were incubated for 30 min at 30 °C in kinase reaction buffer containing 5 µg of dephospho-casein (Sigma), 25 mM Tris-HCl pH 7.5, 2 mM DTT, 10 mM MgCl_2_, 100 µM ATP, 1 µCi ATP_γ_32^P^, 0.5 mM EDTA, 25 µM bromolevamisole oxalate, 5 mM β-glycerophosphate, 1 µM benzamidine, 10 µM pepstatin A, 5 µg/mL leupeptin, 0.2 mM tungstate, and 0.1 mM Na_3_VO_4_. Reaction mixtures were resolved in 13% SDS-polyacrylamide gels. Phosphorylated casein was detected using a Typhoon Trio (GE Healthcare) and quantified using Image J^54^. The effect of Dbf4 was assessed by addition of 4 molar excess of a Dbf4-derived peptide (^73^EKKRARIERARSIEGAVQVSKGTG^96^) to the kinase reactions. Experiments were performed in triplicates (n = 3).

## Supplementary information

Supplementary Information.

## Data Availability

The crystal structures have been deposited on the protein data bank (accession codes 6MF4, 6MF5, and 6MF6).
