# Supplementary material for: Distinct surfaces on Cdc5/PLK Polo-box domain orchestrate combinatorial substrate recognition during cell division
Source: Sci Rep. 2020 Feb 25;10:3379. doi: 10.1038/s41598-020-60344-4 (PMC7042354; doi:10.1038/s41598-020-60344-4)
Supplement: Supplementary file 1 — Supplementary Information. [file 41598_2020_60344_MOESM1_ESM.pdf]

Distinct surfaces on Cdc5/PLK Polo-box domain orchestrate combinatorial substrate recognition during cell division

Ahmad W. Almawi[1,#], Laurence Langlois-Lemay[2], Stephen Boulton[3], Javier Rodriguez Gonzalez[4], Giuseppe Melacini[1,3], Damien D'Amours[2*], and Alba Guarné[1,4*].

Supporting Information:

Supplementary Figure 1: Conserved hydrophobic pocket on polo box 1

Supplementary Figure 2: Polo-box domains have a conserved phosphopeptide binding mode

Supplementary Figure 3: STD spectra of the individual substrates

Supplementary Figure 4: Nucleus and spindle pole body separation phenotypes of the cdc5-S630Q mutant

Supplementary Figure 5: Effect of the Dbf4 peptide on the kinase activity of Cdc5

Supplementary Table 1: STD/STR ratios of the Spc72 peptides

Supplementary Table 2: Yeast strains used in this study

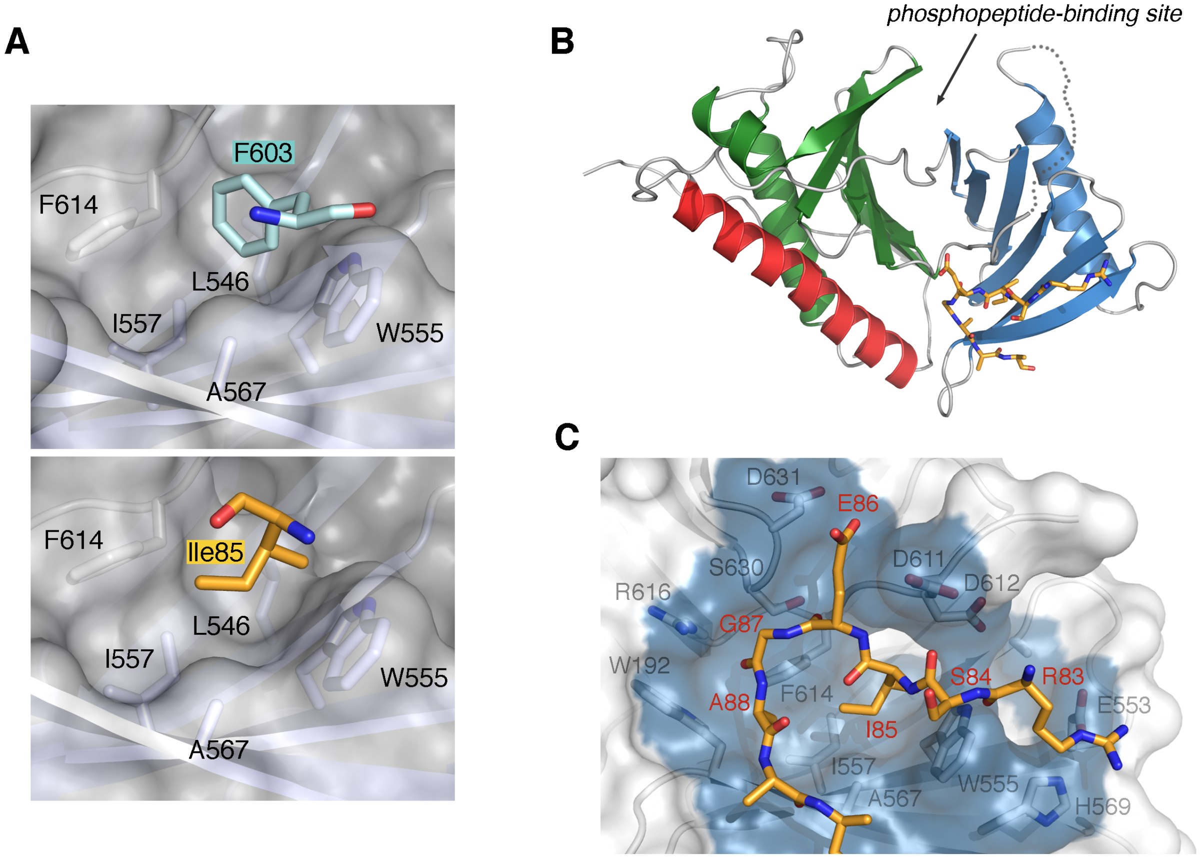

Supplementary Fig. 1. Conserved hydrophobic pocket on polo box 1. a) Detail of the Cdc5 structure with Phe603 residue from the cleaved α2-β7 loop sitting in the hydrophobic pocket defined by Leu546, Trp555, Ile557 and Ala567 (top). Detail of the Cdc5 structure crystallized in the presence of the Dbf4 peptide with Ile85 (from the Dbf4 peptide) modelled based on the difference electron density maps (see **Fig. 3a**). Note that the extra electron density is found at a similar position to that of Phe603 on the crystal of the polo-box domain of Cdc5 on its own (see **Fig.1c**). **b)** Model of the Dbf4 peptide in the structure of Cdc5 bound to Dbf4. **c)** Detail view of the Dbf4-binding pocket with Dbf4 residues (red labels) shown as color coded sticks and Cdc5 residues indicated with grey labels.

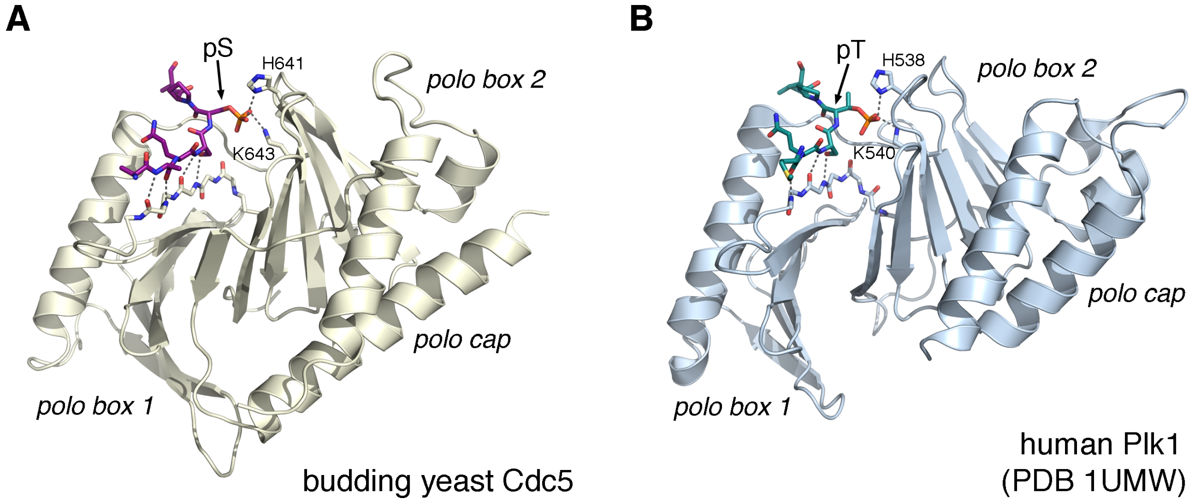

Supplementary Fig. 2. Polo-box domains have a conserved phosphopeptide binding mode. The polo-box domain of S. cerevisiae Cdc5 **(a)** and human Plk1 **(b)** bind target phosphopeptides at the groove defined by the two polo boxes and use conserved His and Lys residues to stabilize the phosphate group.

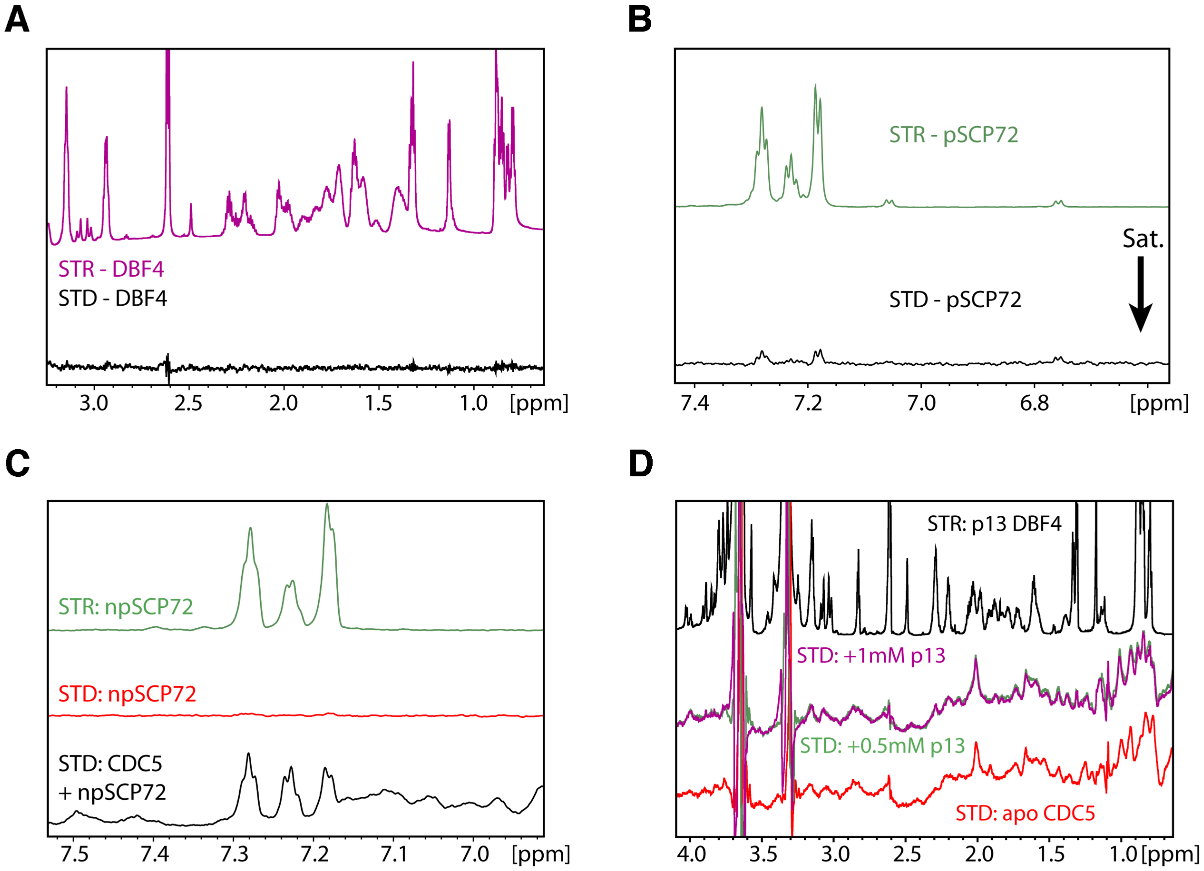

Supplementary Fig. 3. Control STD NMR spectra of the individual substrates. STD spectra of Dbf4 **(a)**, Scp72^{P} **(b)**, and Spc72 **(c)**. **d)** STD spectra of a short Dbf4 (13 amino acids: p13) peptide. The STR spectra and STD spectra of Cdc5 are provided for comparison.

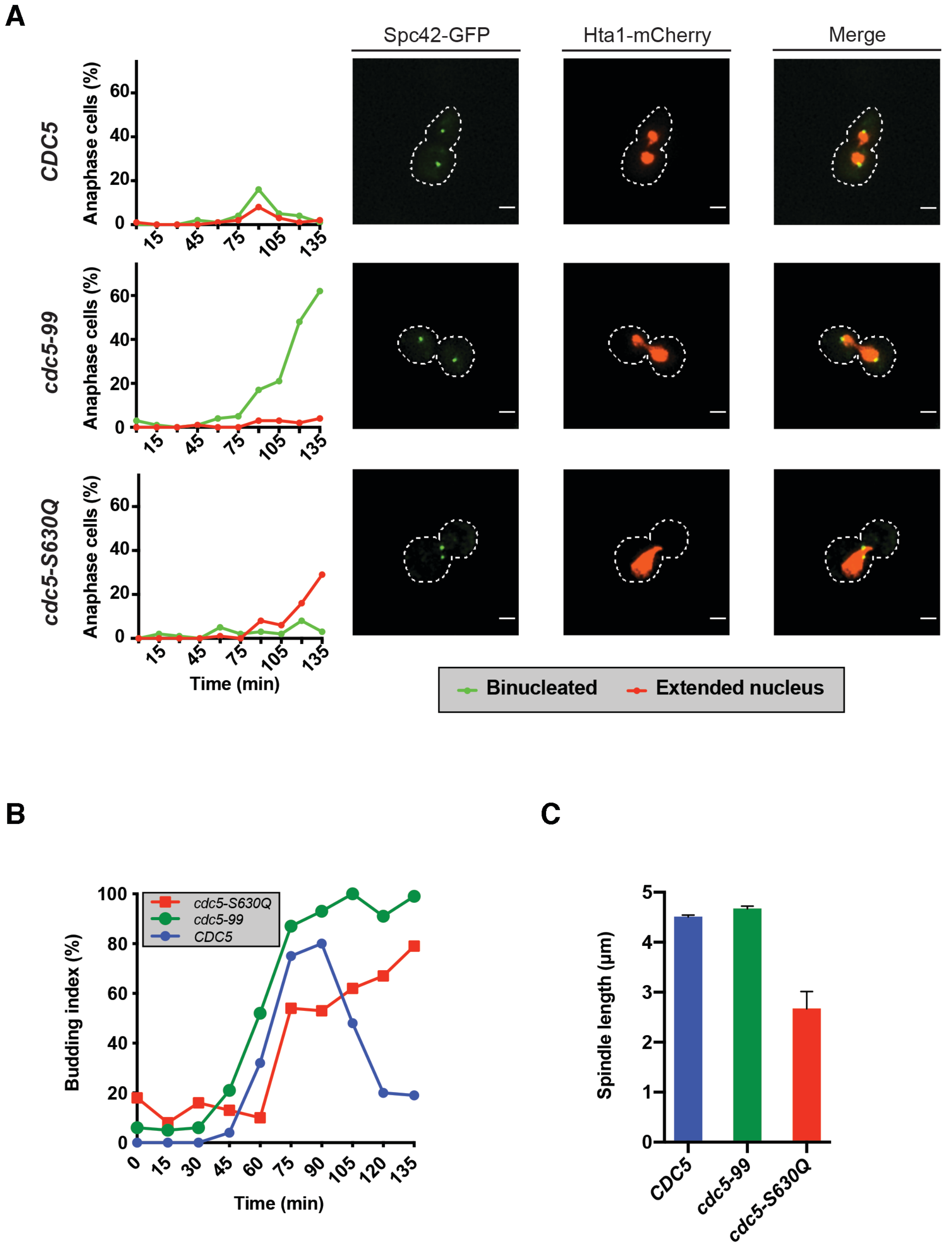

Supplementary Fig. 4. Nucleus and spindle pole body separation phenotypes of the *cdc5-S630Q* mutant. Exponential cultures of *CDC5, cdc5-99* and *cdc5-S630Q* strains expressing Spc42-GFP and Hta1-mCherry were synchronized in G1 using α-factor at 23 °C. After synchronous release from the G1 arrest,

cells were grown at 37 °C and samples collected at regular intervals to monitor the morphology of nuclei and distance separating SPBs by fluorescent microscopy. **(A)** The graphs on the left show a quantification of nuclear morphology for each strain during the time-course experiment, whereas the micrographs on the right show the morphology and position of nuclei/SPBs in mid-anaphase cells. The quantification of nuclear morphology focused on two classes of Hta1-mCherry signal observed during anaphase: binucleated cells with little or no chromatin connecting the nuclear masses or mitotic cells with extended/stretched nuclei in the early stages of chromosome separation. Cell outlines were marked with dotted lines. Bar is 2 µm. **(B)** Kinetics of bud formation (*i.e.*, budding index) in cultures of yeast described in panel (A). At least 100 cells were counted at each time point. Note that we observed *cdc5-S630Q* cells expressing Spc42-GFP and Hta1-mCherry markers progressed in the cell cycle with a slight delay relative to mutants carrying wild-type *SPC42* and *HTA1*, possibly reflecting a synthetic interaction between the *CDC5* allele and fluorescent markers. **(C)** Analysis of spindle length in mid-anaphase cells expressing *CDC5, cdc5-99,* and *cdc5-S630Q* mutants. To measure the length of the nuclear spindle in yeast, the distance separating Spc42-GFP foci was determined in at least 30 anaphase cells, as previously described (Maddox *et al* (2012) *Methods Enzymol.* 505:81-103). Error bars represent SEM (n=3).

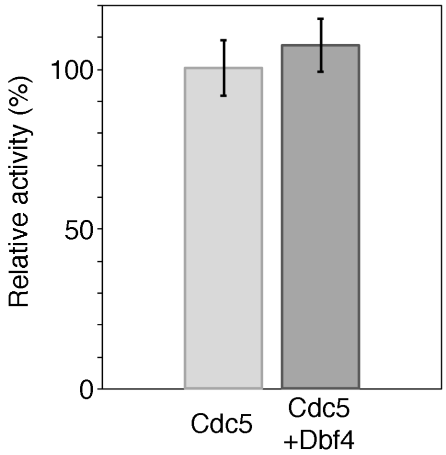

Supplementary Fig. 5. Effect of the Dbf4 peptide on the kinase activity of Cdc5. (A) The kinase activity of Cdc5 (0.125 pmol) was tested as previously described (Ratsima *et al* (2011) *PNAS* 108(43), E914-E923) in the presence/absence of the Dbf4 peptide (^{73}EKKRARIERARSIEGAVQVSKGTG^{96}). Error bars represent SEM over 3 measurements.

Supplemental Table 1: STD/STR ratios of the Spc72 peptides.

Sample	STD/STR (1)* ($x10^{-3}$)	STR/STR (2) ($x10^{-3}$)	STD/STR (3) ($x10^{-3}$)
**Spc72^{P}**	9±1	7±2	7±1
**Spc72**	9±3	N/D	8±2
**Cdc5-Spc72^{P}**	97.0±0.5	125±1	50.4±0.4
**Cdc5-Spc72**	70±2	107±3	38±1
**Dbf4-Cdc5-Spc72^{P}**	89.8±0.5	114±1	50.5±0.4

**The STD/STR ratios were calculated for the three peaks in Figure 5B*

Supplemental Table 2: Yeast strains used in this study.

	Strain #	Relevant genotype
**Figure 6**	D4107	*MAT**a** CDC5*
	D1224	*MAT**a** cdc5-77::HIS3MX6*
	D224	*MAT**a** smc5-6::HIS3*
	D6502	*MAT**a** cdc5-H569A::HIS3MX6*
	D6460	*MAT**a** cdc5-R616A::HIS3MX6*
	D6462	*MAT**a** cdc5-S630A::HIS3MX6*
	D6505	*MAT**a** cdc5-S630Q::HIS3MX6*
	D6507	*MAT**a** cdc5-A567W::HIS3MX6*
**Figure 7**	D4107	*MAT**a** CDC5*
	D777	*MAT**a** cdc5-99::HIS3MX6*
	D6462	*MAT**a** cdc5-S630A::HIS3MX6*
	D6505	*MAT**a** cdc5-S630Q::HIS3MX6*
